# An Unusual Case Report of Erupted Odontoma

**DOI:** 10.1155/2013/570954

**Published:** 2013-02-05

**Authors:** Dhaval Mehta, Nilesh Raval, Sneha Udhani, Viral Parekh, Chintan Modi

**Affiliations:** Department of Oral Medicine and Radiology, Karnavati School of Dentistry, 907/A Uvarsad, Gandhinagar, Gujarat 382422, India

## Abstract

Odontomas are the most common of the odontogenic tumors of the jaws, which are benign, slow growing, and nonaggressive. They are usually asymptomatic and found in routine dental radiographic examination. Odontomas are usually associated with tooth eruption disturbances. Eruption of odontoma in oral cavity is rare entity. Here we report a case of an unusual erupted compound odontoma.

## 1. Introduction

Odontomas are hamartomas composed of various dental tissues, that is, enamel, dentin, cementum, and sometimes pulp. They are slow-growing, benign tumours. Paul Broca was the first to coin the term “odontoma” in 1867. The World Health Organisation (WHO) defines odontomas as two types: complex odontomas, a malformation in which all dental tissues are present, but arranged in a more or less disorderly pattern; and compound odontomas, a malformation in which all of the dental tissues are represented in a pattern that is more orderly than that of the complex type [1]. The erupted odontomas are the ones which are present coronal to an erupting or impacted tooth or superficially in bone and may have enabled its eruption into the oral cavity [2]. Odontomas erupting into the oral cavity are extremely uncommon, with the first case being reported in English literature in 1980 by Rumel et al. A literature review by Litonjua et al. in 2003 recorded only 14 cases in English literature from 1980 to 2003 [2, 3]. To date, 20 cases of erupted odontomas are reported in the literature; out of these 11 cases are complex odontoma and 9 erupted odontomas are associated with impacted teeth [4].

Here we present a rare case of erupted odontome in maxillary anterior region.

## 2. Case Report

A 15-year old female patient came with complain of presence of a small hard mass in upper front tooth region and its undesirable appearance was a concern. She had no complaint of pain or any other previous infection.

**Figure 1:**
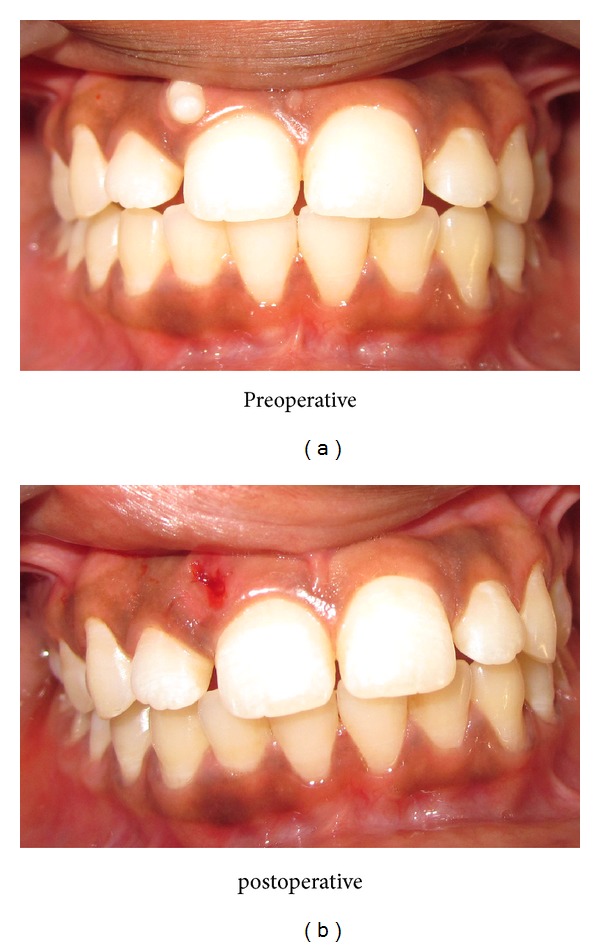
Compound odontoma in right upper maxillary central incisor region (preoperative and postoperative).

**Figure 2:**
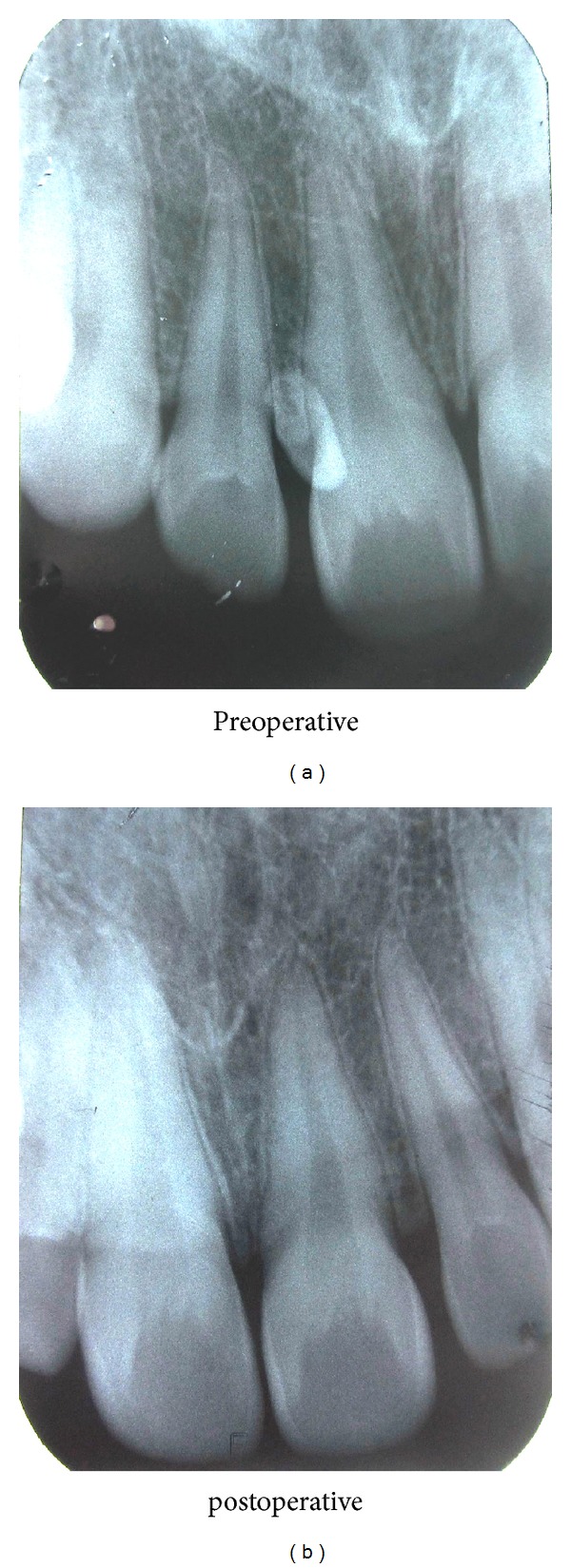
IOPA showing tooth-like radiopaque structure in right upper maxillary central incisor region suggestive of erupted compound odontoma (preoperative and postoperative).

Patient was fit and well medically. Intraoral examination revealed a small whitish oval tooth like structure present in maxillary anterior region. It is placed superiorly and labially in attached gingival in relation to crown of right maxillary central incisor region (Figure 1). It was hard, nontender, and grade II mobile. Neither expansion of any cortical plates (Figure 2) nor any pus discharge present.

Intraoral periapical radiograph revealed a well-defined radiopaque mass similar to density of dental tissues. It was placed apical to crown of right maxillary central incisor and in interdental bone between maxillary right central and lateral incisors suggesting erupted compound odontoma. Removal of odontoma was done and sent for histopathological report.

**Figure 3:**
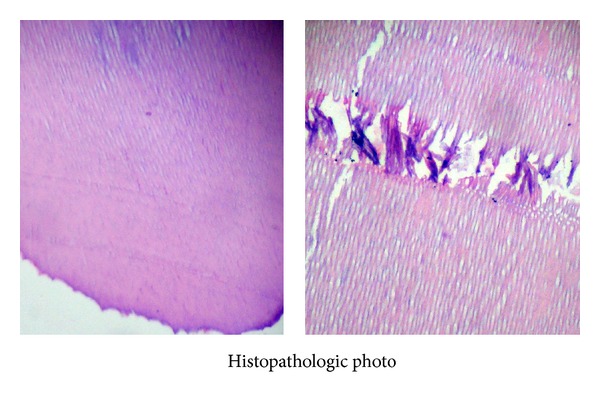
Decalcified section showing organized mass of enamel, dentine, and pulp tissue.

Histopathological examination confirmed the specimen to be compound odontoma consisting of organized mass of enamel, dentine, and pulp tissue resembling the structure of tooth (Figure 3).

## 3. Discussion 

Odontoma is the most common odontogenic tumor in maxilla. Compound odontoma most commonly found in the upper incisors and canines areas, followed by the antero- and posteroinferior regions. Complex odontomas are commonly found in the areas of the second and third lower molars. An increased prevalence of these tumors is observed in children and adolescents. These lesions are normally diagnosed by routine radiological features in the second and third decades of life [5].

Hitchin suggested that odontomas are inherited through a mutant gene or interference, possibly postnatal, with genetic control of tooth development. In humans, there is a tendency for the lamina between the tooth germs to disintegrate into clumps of cells. The persistence of a portion of lamina may be an important factor in the etiology of complex or compound odontomas and either of these may occur instead of a tooth [6].

Odontomas have been associated with trauma during primary dentition, as well as with inflammatory and infectious processes, hereditary anomalies (Gardner syndrome and Hermann syndrome), and odontoblastic hyperactivity, and alterations in the genetic components are responsible for controlling dental development [2].

Usually odontomas are asymptomatic. Clinical indicators of odontoma may include retention of deciduous teeth, noneruption of permanent teeth, pain, expansion of cortical bone, and tooth displacement. Pain and swelling are the most common symptoms when odontomas erupt, followed by malocclusion. Some odontomas appear at very young age and others at old age [3].

Although the odontomas erupting in the oral cavity are controversial, the reason is attributed to the eruptive forces of the apparently impacted teeth, and in cases of absence of teeth, the reasons could possibly be the resorption of alveolar ridge exposing the odontoma, sequestration of overlying bone, alveolar bone remodeling in young adults, or reactive growth of the capsule surrounding odontoma in elderly patients [2, 3].

The mechanism of odontoma eruption appears to be different from tooth eruption because of the lack of periodontal ligament and root in odontoma. Therefore the force required to move the odontoma is not linked to the contractility of the fibroblasts, as in the case for teeth. Although there is no root formation in odontoma, its increasing size may lead to the sequestration of the overlying bone and, hence, occlusal movement or eruption. An increase in the size of the odontoma over time produces a force sufficient to cause bone resorption [4].

The odontoma presents as a well-defined radiopacity situated in bone, but with a density that is greater than bone and equal to or greater than that of a tooth. It contains foci of variable density. A radiolucent halo, typically surrounded by a thin sclerotic line, surrounds the radiopacity. The radiolucent zone is the connective tissue capsule of a normal tooth follicle. The thin sclerotic line resembles the corticated border seen in a normal tooth crypt. The developmental stages can be identified based on radiologic features and the degree of calci-fication of the lesion at the time of diagnosis [7]. The first stage is characterized by radiolucency due to the absence of dental tissue calcification, the second or intermediate stage shows partial calcification, and the third or classically radiopaque stage exhibits predominant tissue calcification with the surrounding radiolucent halo described above [8].

Removal of the lesion and enveloping soft tissue with curettage to prevent cystic degeneration is treatment of choice. In case of impacted tooth with compound odontoma, surgical removal of odontoma with orthodontic eruption of impacted tooth is indicated.

## 4. Conclusion

A rare case of erupted compound odontoma that was about to be exfoliated has been reported. Odontomas rarely erupt into the mouth and tend to be associated with impacted teeth. Despite their benign nature, however, their eruption into the oral cavity can give rise to pain, inflammation, and infection and different clinical appearance.

## References

[1] Philipsen HP, Reichart PA, Prætorius F (1997). Mixed odontogenic tumours and odontomas. Considerations on interrelationship. Review of the literature and presentation of 134 new cases of odontomas. *European Journal of Cancer Part B*.

[2] Ragalli CC, Ferreria JL, Blasco F (2000). Large erupting complex odontoma. *International Journal of Oral and Maxillofacial Surgery*.

[3] Vengal M, Arora H, Ghosh S, Pai KM (2007). Large erupting complex odontoma: a case report. *Journal of the Canadian Dental Association*.

[4] Serra-Serra G, Berini-Aytés L, Gay-Escoda C (2009). Erupted odontomas: a report of three cases and review of the literature. *Medicina Oral, Patologia Oral y Cirugia Bucal*.

[5] Budnick SD (1976). Compound and complex odontomas. *Oral Surgery Oral Medicine and Oral Pathology*.

[6] Hitchin AD (1971). The aetiology of the calcified composite odontomes. *British Dental Journal*.

[7] Junquera L, De Vicente JC, Roig P, Olay S, Rodríguez-Recio O (2005). Intraosseus odontoma erupted into the oral cavity: an unusual pathology. *Medicina Oral, Patologia Oral y Cirugia Bucal*.

[8] Guinta JL, Kaplan MA (1990). Peripheral soft tissue odontomas. *Oral Surgery, Oral Medicine, Oral Pathology*.

